# Reversal of MRP7 (ABCC10)-Mediated Multidrug Resistance by Tariquidar

**DOI:** 10.1371/journal.pone.0055576

**Published:** 2013-02-05

**Authors:** Yue-Li Sun, Jun-Jiang Chen, Priyank Kumar, Kang Chen, Kamlesh Sodani, Atish Patel, Yang-Lu Chen, Si-Dong Chen, Wen-Qi Jiang, Zhe-Sheng Chen

**Affiliations:** 1 State Key Laboratory of Oncology in South China, Guangzhou, Guangdong, People's Republic of China; 2 Department of Medical Oncology, Sun Yat-sen University Cancer Center, Guangzhou, Guangdong, People's Republic of China; 3 Department of Pharmaceutical Sciences, College of Pharmacy and Health Sciences, St. John's University, Queens, New York, United States of America; 4 Guangdong Key Laboratory for Molecular Epidemiology, School of Public Health, Guangdong Pharmaceutical University, Guangzhou, Guangdong, People's Republic of China; 5 Department of Obstetrics and Gynecology, Wayne State University School of Medicine, Detroit, Michigan, United States of America; 6 Perinatology Research Branch, Eunice Kennedy Shriver National Institute of Child Health and Human Development, National Institutes of Health, Bethesda, Maryland, United States of America; 7 Montgomery High School, Skillman, New Jersey, United States of America; Indiana University School of Medicine, United States of America

## Abstract

Multidrug resistance protein 7 (MRP7, ABCC10) is a recently discovered member of the ATP-binding cassette (ABC) family which are capable of conferring resistance to a variety of anticancer drugs, including taxanes and nucleoside analogs, *in vivo*. MRP7 is highly expressed in non-small cell lung cancer cells, and *Mrp7-KO* mice are highly sensitive to paclitaxel, making MRP7 an attractive chemotherapeutic target of non-small cell lung cancer. However, only a few inhibitors of MRP7 are currently identified, with none of them having progressed to clinical trials. We used MRP7-expressing cells to investigate whether tariquidar, a third generation inhibitor of P-glycoprotein, could inhibit MRP7-mediated multidrug resistance (MDR). We found that tariquidar, at 0.1 and 0.3 µM, significantly potentiated the sensitivity of *MRP7*-transfected HEK293 cells to MRP7 substrates and increased the intracellular accumulation of paclitaxel. We further demonstrated that tariquidar directly impaired paclitaxel efflux and could downregulate MRP7 protein expression in a concentration- and time-dependent manner after prolonged treatment. Our findings suggest that tariquidar, at pharmacologically achievable concentrations, reverses MRP7-mediated MDR through inhibition of MRP7 protein expression and function, and thus represents a promising therapeutic agent in the clinical treatment of chemoresistant cancer patients.

## Introduction

Multidrug resistance (MDR) against chemotherapeutic regimens remains a major roadblock to the successful treatment of cancer. For the past three decades, the mechanisms underlying MDR have been extensively studied, wherein active efflux mediated by drug efflux pumps has been considered as a major factor [1]. The ATP-binding cassette (ABC) proteins represent a highly diversified superfamily with functions ranging from ion transport to macromolecular efflux [2]. By using the energy derived from ATP hydrolysis, ABC transporters could extrude a wide variety of structurally and functionally unrelated substrates from cells, leading to decreased intracellular concentrations of these substrates and thereby inducing MDR [3]. To date, 49 different ABC transporters have been identified in humans, 14 of which are associated with various diseases [4], [5]. Differences are observed in their functions, substrate specificities, molecular mechanisms and *in vivo* localization [6]. Based on the sequence similarity as well as structural organization, ABC transporters are divided into seven subfamilies [7]. Among them, P-glycoprotein (P-gp, ABCB1), multidrug resistance proteins (MRPs, ABCCs) and breast cancer resistant protein (BCRP, ABCG2) are widely studied. ABCCs consist of 13 members, and 9 of them are related to MDR. Therefore, they are called MRPs [8]. In the present study, we focus on a member of the MRP subfamily, MRP7. Compared with MRP1, relatively little information is available about MRP7. MRP7 was shown to have the ability to mediate the transport of estradiol 17-β-D-Glucuronide (E_2_17βG), and to a lesser extent, leukotriene C4 (LTC4) [9], [10], but not other MRP substrates such as cyclic nucleotides, methotrexate or bile acids *in vitro* [10]. Unlike other MRPs, MRP7 was capable of conferring resistance to taxanes [9], and was demonstrated to be used as a predictive marker of resistance to paclitaxel in non-small cell lung cancer (NSCLC) [11]. Recently, it is reported that MRP7 can affect the *in vivo* tissue sensitivity towards taxanes, the widely used anticancer drugs in NSCLC, independently of P-gp [12].

Overcoming ABC transporter-mediated MDR can be achieved by interfering with the expression of the transporter proteins or their functions. It was speculated that inhibiting these transporters would restore the cytotoxicity of available anticancer drugs against resistant cells. A significant number of compounds have been identified to reverse ABC transporter-mediated MDR [13]. Presently, three generations of P-gp modulators have been developed to increase the sensitivity of chemotherapeutic drugs in MDR cancer cells [14]. A variety of inhibitors of BCRP have also been identified and classified into four categories: 1) BCRP-specific inhibitors, 2) compounds that also inhibit P-gp and/or MRP1, 3) naturally occurring flavonoids and derivatives and 4) tyrosine kinase inhibitors (TKI) [15]. However, the development of most of these inhibitors has been discontinued due to low binding affinity, toxicity, detrimental pharmacokinetic interactions and low patient survival advantages [3], [16], [17]. In addition, very few reversal agents for MRP members have been discovered or progressed to clinical trials. Therefore, there is a continuous need for the discovery of potent and specific inhibitors of MRP transporters.

**Figure 1:**
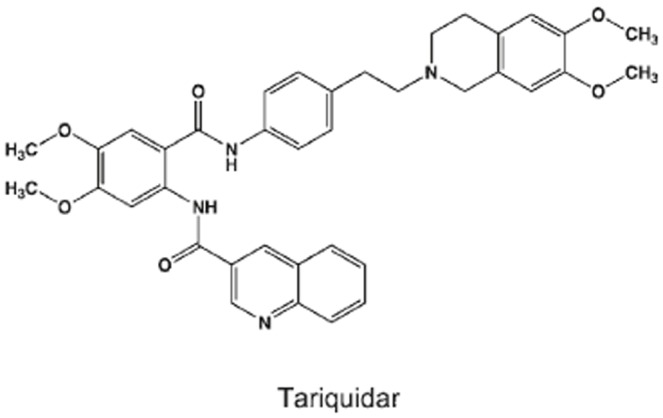
Structure of tariquidar (XR9576).

Tariquidar (XR9576, the chemical structure is shown in **Figure 1**) is a third generation P-gp inhibitor with high affinity (*K*_d_ = 5 nM), low toxicity and increased selectivity [18], [19]. Tariquidar was considered as an ideal agent for testing the role of P-gp inhibition in cancer [20]. Later, it was also found to be an inhibitor of BCRP [21], [22], but had no interaction with MRP1 [22], indicating that this inhibitor may not be as specific as previously thought. However, whether tariquidar can interact and inhibit other MRP members is unknown. In the present study, we investigated the ability of tariquidar to reverse MRP7-mediated MDR. We found that tariquidar could significantly increase the cellular sensitivity to MRP7 substrates. Mechanistically, tariquidar could increase the intracellular concentration of [^3^H]-paclitaxel by inhibiting the efflux function of MRP7 pump and decrease the expression of MRP7 protein without altering mRNA level.

## Materials and Methods

### Chemicals

Paclitaxel, docetaxel, vincristine, vinblastine, vinorelbine, dimethyl sulfoxide (DMSO) and 1-(4,5-dimethylthiazol-2-yl)-3,5-diphenylformazan (MTT) were purchased from Sigma-Aldrich (St. Louis, MO). Tariquidar was kindly given by Drs. Susan E. Bates and Robert W. Robey (NCI, NIH). Cepharanthine was generously provided by Kakenshoyaku Co. (Tokyo, Japan). [^3^H]-paclitaxel (38.9 Ci/mmol) was purchased from Moravek Biochemicals (Brea, CA). Boron dipyrromethene (BODIPY)-conjugated paclitaxel was purchased from Life technologies (Grand Island, NY).

### Cell lines and cell culture

The previously reported MRP7 expression vector and parental plasmid [10] were transfected into HEK293 cells by electroporation. Transfected cells were selected in DMEM containing 2 mg/ml G418. The parental cell line HEK293 transfected with empty vector was represented as HEK/pcDNA. And HEK293 transfected with MRP7 expression vector cell line was represented as HEK/MRP7. MRP7 protein was detected by immunoblotting. All cell lines were grown as adherent monolayers in DMEM supplemented with 10% fetal bovine serum (FBS), 200 U/ml penicillin and 200 U/ml streptomycin (Hyclone, Logan, UT) in a 5% CO_2_ incubator at 37°C.

### MTT assay

An MTT colorimetric assay with minor modifications from that previously described [23] was used to detect the sensitivity of cells to anticancer drugs. Cells were harvested with trypsin treatment and resuspended at a concentration of 6×10^3^ cells/well. For the reversal experiment, tariquidar (0.1 µM or 0.3 µM, 20 µl/well) or cepharanthine (2.5 µM, 20 µl/well) was added followed by different concentrations of chemotherapeutic drugs (20 µl/well) into designated wells. After 68 h of incubation, 20 µl of MTT solution (4 mg/ml) was added to each well, and the plate was further incubated for another 4 h, allowing viable cells to convert the yellow-colored MTT into dark blue formazan crystals. Subsequently, the medium was aspirated, and 100 µl DMSO was added to each well to dissolve the formazan crystals. The absorbance was determined at 570 nm and 630 nm by an OPSYS Microplate Reader (DYNEX Technologies, Chantilly, VA). The degree of resistance was calculated by dividing the IC_50_ (concentrations required to inhibit growth by 50%) for the MDR cells by that of the parental sensitive cells. The IC_50_ values were calculated to construct the survival curves using the Bliss method [24].

### Immunoblotting

Total cell lysates were prepared by harvesting the cells and rinsing three times with ice-cold PBS. Cell extracts were prepared by incubating cells for 30 min on ice with radioimmunoprecipitation assay (RIPA) buffer (PBS with 0.1% SDS, 1% Nonidet P-40, 0.5% sodium deoxycholate, and 100 mg/ml p-aminophenylmethylsulfonyl fluoride) with occasional rocking followed by centrifugation (12,000 g, 4°C for 20 min). The supernatant containing total cell lysates was stored at −80°C prior to experiments. Cell lysates containing identical amounts of total protein (40 µg) were resolved by sodium dodecyl sulfate polycrylamide gel electrophoresis (SDS-PAGE) and transferred onto polyvinylidene fluoride (PVDF) membranes. After incubation in a blocking TBST buffer (10 mmol/L Tris-HCl (PH 8.0), 150 mmol/L NaCl, and 0.1% Tween 20, and 5% non-fat milk) for 2 h at room temperature, the membranes were immunoblotted overnight with primary antibodies against MRP7 (1∶200 dilution) (Santa Cruz Biotechnology, Santa Cruz, CA) or GAPDH (1∶1000 dilution) (Cell Signaling, Danvers, MA) at 4°C, and then incubated at room temperature with horseradish peroxidase (HRP)-conjugated secondary antibody (1∶1000 dilution) for 2 h. The protein-antibody complex was detected by chemoluminescence. Densitometry analysis was performed using the Quantity One software (Bio-Rad, Hercules, CA). MRP7 immunoblots were normalized to GAPDH immunoblots for each sample analyzed.

### [^3^H]-paclitaxel accumulation and efflux

Intracellular paclitaxel accumulation and efflux were measured in HEK/pcDNA cells and HEK/MRP7 cells. For 4 h accumulation assay, the cells were trypsinized and three aliquots (5×10^6^ cells) from each cell line were resuspended in the medium. To measure drug accumulation, cells were preincubated in DMEM in the presence or absence of tariquidar (at 0.1 and 0.3 µM) or cepharanthine (at 2.5 µM) for 2 h, washed and then incubated with 0.01 µM [^3^H]-paclitaxel with or without tariquidar (at 0.1 µM and 0.3 µM) or cepharanthine (at 2.5 µM) for another 2 h at 37°C. The cells were pelleted at 4°C, washed twice with 10 ml ice-cold PBS, and lysed in 1% SDS. Radioactivity was measured in a liquid scintillation counter. For 72 h accumulation assay, cells were cultured in DMEM in the absence or presence of tariquidar for 70 h. Cells were then trypsinized and resuspended with the same cell number in each group, and incubated with 0.01 µM [^3^H]-paclitaxel with or without tariquidar (at 0.3 µM) for another 2 h at 37°C. After this, the steps were the same as 4 h accumulation. For the efflux study, cells were incubated with 0.01 µM [^3^H]-paclitaxel according to the method for the accumulation study. After being washed twice with cold PBS, the cells were cultured in fresh DMEM with or without 0.3 µM tariquidar at 37°C. After 0, 30, 60 or 120 min, aliquots of cells were removed and immediately washed with ice-cold PBS. The cell pellets were collected for radioactivity measurement in a Packard TRI-CARB® 1900CA liquid scintillation analyzer (Packard Instrument Company, Downers Grove, IL).

### BODIPY-paclitaxel accumulation assay

After culturing in the presence or absence of tariquidar for 4 h or 72 h, HEK/pcDNA and HEK/MRP7 cells were harvested and transferred to DMEM/FBS containing 40 ng/mL BODIPY-paclitaxel. After 30 min incubation at 37°C, cells were pelleted and resuspended in medium with or without tariquidar in addition to BODIPY-paclitaxel. One hour later, cells were pelleted, resuspended, and immediately analyzed on an LSRFortessa flow cytometer (BD Biosciences). 7-Aminoactinomycin D (7-AAD) (BD Biosciences) was used to exclude non-viable cells from analysis.

### Reverse transcription polymerase chain reaction (RT-PCR)

Total RNA was extracted from HEK/pcDNA and HEK/MRP7 cells using Trizol (Invitrogen, Grand Island, NY) according to manufacturer's instructions. Reverse transcription was performed with 2 µg total RNA in a volume of 20 µl by Transcription System (Promega, Madison, WI) according to the manufacturer's instructions. The sequences of the *MRP7* and *GAPDH* primers were as follows: *MRP7* (303 bp) sense: 5′-GGCTCCGGCAAGTCTTCCCTGTT-3′ and antisense: 5′-AGATAAGCTCCGGCCCCCCTCACC-3′. *GAPDH* (322 bp) sense: 5′-CGGGAAGCTTGTCATCAATGG-3′ and antisense 5′-GGCAGTGATGGCATGGACTG-3′. PCR was carried out with GoTaq® Master Mixes and the reaction conditions were 94°C for 30 s, 60°C for 40 s (*GAPDH*) or 65°C for 40 s (*MRP7*), 72°C for 45 s with 35 cycles. The PCR products were separated by agarose gel electrophoresis. The gel was stained with 0.5 µg/ml ethidium bromide, and the bands were visualized under UV light.

### Immunofluorescence

Cells (1×10^4^) were seeded in 24-well plate and tariquidar at 0.3 µM was added into the wells at different time points after overnight culture. After 72 h, cells were washed with PBS and fixed with 4% paraformaldehyde for 15 min at room temperature and then rinsed with PBS three times. Non-specific reaction was blocked with BSA (2 mg/ml) for 1 h at 37°C. A polyclonal antibody against MRP7 (D19) (1∶50) (Santa Cruz Biotechnology, Santa Cruz, CA) was applied overnight, followed by an Alexa flour 488-conjugated goat anti-mouse IgG (1∶100) (Molecular Probes, Eugene, OR) for 1 h. DAPI was used to counterstain the nuclei. Images were taken with an inverted IX70 microscope (Olympus, Center Valley, PA) with IX-FLA fluorescence and CCD camera.

### Statistical analysis

All experiments were repeated for at least three times. Statistical differences were determined by the two-tailed Student's t-test, and were deemed significant if *P<0.05*.

## Results

### Tariquidar potentiates the sensitivity of anticancer drugs in MRP7-expressing cells

Before determining the MDR reversal effects of tariquidar, we first examined its effect on the cell viability of HEK/pcDNA and HEK/MRP7 cell lines with MTT assay. The non-cytotoxic concentration of tariquidar in HEK/pcDNA and HEK/MRP7 cell lines were more than 3 µM (data not shown). However, to avoid cytotoxicity, the highest concentration of tariquidar used in the subsequent studies was 0.3 µM, which induced less than 10% growth inhibition in the two cell lines.

**Figure 2:**
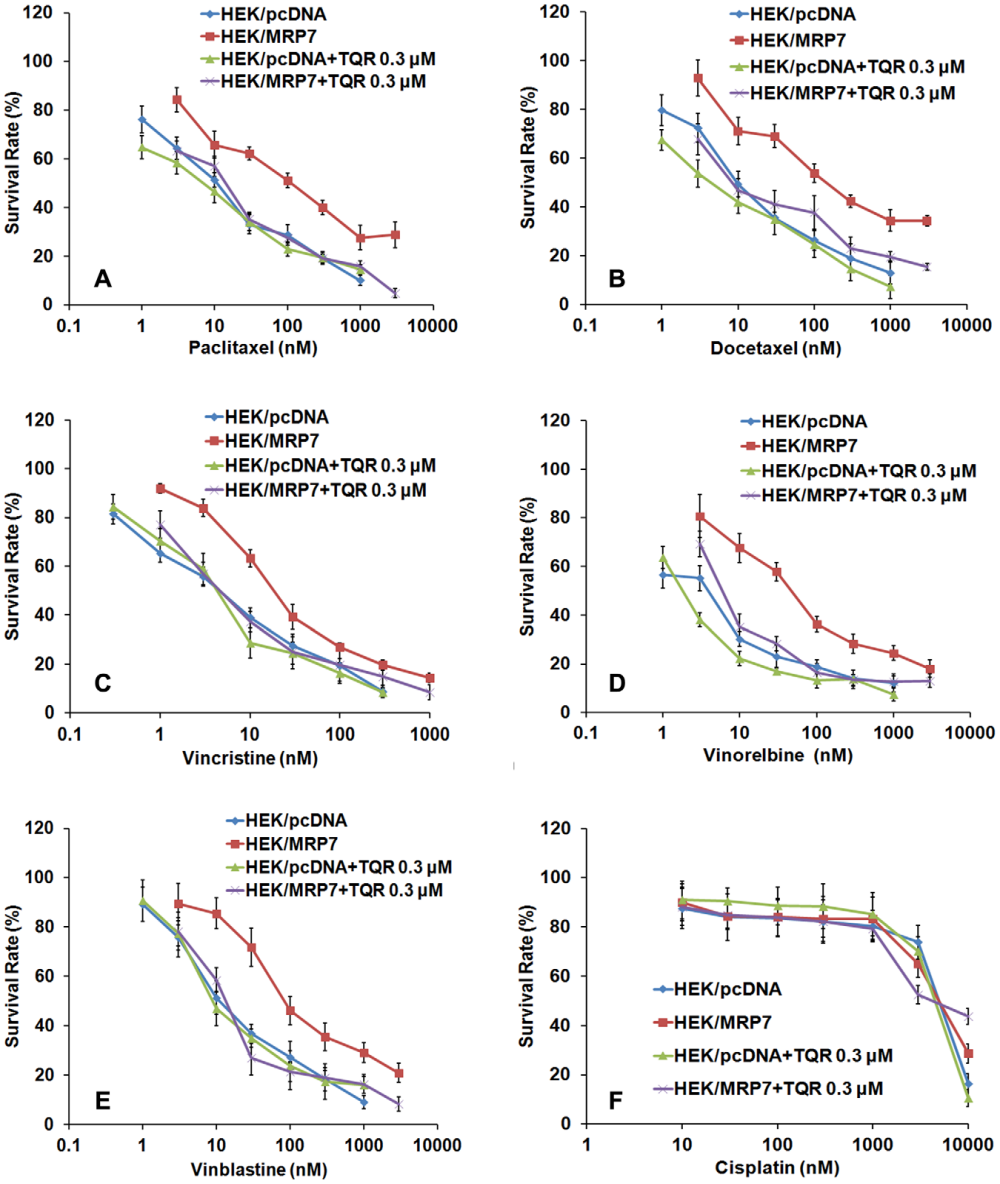
Effect of tariquidar on the sensitivity of HEK/pcDNA and HEK/MRP7 cells to anticancer drugs. HEK/pcDNA cells and HEK/MRP7 cells were cultured for 24 h before tariquidar was added. After 1 h incubation, paclitaxel (A), docetaxel (B), vincristine (C), vinorelbine (D), vinblastine (E), or cisplatin (F) was added, and the cultures were incubated for 72 h. Cell viability was measured by the MTT assay. Results were representative of 3 independent experiments.

Subsequently, we analyzed whether tariquidar could reverse MRP7-mediated drug resistance to anticancer drugs. The IC_50_ values of the chemotherapeutic drugs used in both cell lines in the presence or absence of tariquidar are summarized in **Table 1**. As compared to parental HEK/pcDNA cells, HEK/MRP7 cells exhibited a significant resistance to various MRP7 substrates, including paclitaxel (12.11-fold), docetaxel (17.18-fold), vincristine (4.12-fold), vinblastine (6.7-fold) and vinorelbine (5.9-fold). Tariquidar, at concentrations of 0.1 and 0.3 µM, significantly decreased the IC_50_ values for paclitaxel, docetaxel, vincristine, vinblastine and vinorelbine, which was comparable with cepharanthine at 2.5 µM. Moreover, the IC_50_ values of the aforementioned chemotherapeutic drugs in parental HEK/pcDNA cells showed no significant difference in the presence or absence of tariquidar. Meanwhile, tariquidar did not significantly alter the IC_50_ values of cisplatin, which is not a substrate of MRP7. Such effect of tariquidar was more evident when the growth curves of HEK/MRP7 and HEK/pcDNA were constructed, showing a marked increase in the sensitivity of HEK/MRP7, but not the control HEK/pcDNA cells, specifically to MRP7 substrates in the presence of 0.3 µM tariquidar (**Figure 2**). These results demonstrate that tariquidar significantly potentiates the sensitivity of MRP7 substrates in MRP7-expressing cells.

**Table 1:** Effect of tariquidar and cepharanthine on the cytotoxicity of paclitaxel, docetaxel, vincristine, vinblastine, vinorelbine and cisplatin in *MRP7*-transfected cells.

Drugs	IC_50_±SD^a^ (nM) (Resistance fold)	
	HEK/pcDNA	HEK/MRP7
**Paclitaxel**	8.64±0.99 (1.00)^b^	104.59±13.37 (12.11)
+tariquidar 0.1 µM	8.60±1.41 (1.00)	12.31±2.88 (1.42)*
+tariquidar 0.3 µM	8.81±0.46 (1.02)	8.56±0.57 (0.99)*
+cepharanthine 2.5 µM	9.82±1.81 (1.14)	10.30±1.80 (1.19)*
**Docetaxel**	8.69±0.45 (1.00)^b^	149.31±7.48 (17.18)
+tariquidar 0.1 µM	8.70±0.57 (1.00)	25.64±4.23 (2.95)*
+tariquidar 0.3 µM	8.28±0.28 (0.95)	8.71±1.01 (1.00)*
+cepharanthine 2.5 µM	7.9±1.65 (0.91)	7.44±1.39 (0.86)*
**Vincristine**	5.75±1.17 (1.00)^b^	23.17±3.88 (4.12)
+tariquidar 0.1 µM	6.30±0.52 (1.10)	9.39±0.69 (1.63)*
+tariquidar 0.3 µM	5.47±1.26 (0.95)	6.59±2.05 (1.15)*
+cepharanthine 2.5 µM	5.12±0.55 (0.89)	8.45±0.81 (1.47)*
**Vinblastine**	11.83±1.73 (1.00)^b^	79.28±7.42 (6.70)
+tariquidar 0.1 µM	8.43±0.48 (0.71)	17.26±4.12 (1.46)*
+tariquidar 0.3 µM	7.31±1.06 (0.62)	7.05±0.98 (0.60)*
+cepharanthine 2.5 µM	8.78±0.6 (0.74)	9.52±3.21(0.81)*
**Vinorelbine**	8.62±1.27 (1.00)^b^	50.83±1.54 (5.90)
+tariquidar 0.1 µM	8.47±1.17 (0.98)	18.49±2.34 (2.15)*
+tariquidar 0.3 µM	7.77±0.94 (0.90)	8.00±0.98 (0.93)*
+cepharanthine 2.5 µM	6.73±0.80 (0.78)	8.89±1.06 (1.03)*
**Cisplatin**	4184.29±181.37 (1.00)^b^	5851.14±98.54 (1.40)
+tariquidar 0.1 µM	4731.08±74.72 (1.03)	5099.94±79.53 (1.31)
+tariquidar 0.3 µM	4486.87±58.21 (1.13)	5108.20±103.31 (1.22)
+cepharanthine 2.5 µM	4298.05±244.27 (1.07)	5438.26±130.53 (1.22)

a Values represent mean ± SD of at least three independent experiments, each performed in triplicate.

b Fold of resistance was calculated as the IC_50_ values of paclitaxel, docetaxel, vincristine, vinblastine, vinorelbine or cisplatin of HEK/pcDNA or HEK/MRP7 cells in the absence or presence of reversal agents divided by the IC_50_ values of paclitaxel, docetaxel, vincristine, vinblastine, vinorelbine or cisplatin of HEK/pcDNA cells without the reversing agents.

* : *P<0.05*.

### Tariquidar downregulates MRP7 protein expression without altering MRP7 mRNA level

**Figure 3:**
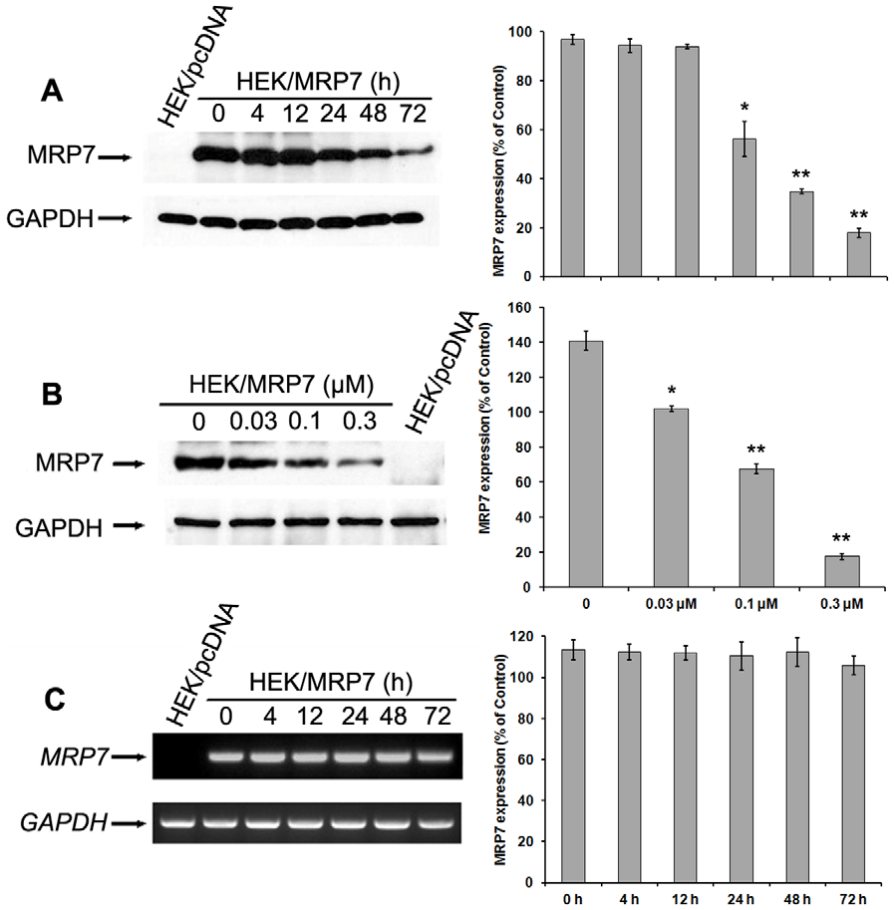
Effect of tariquidar treatment on protein and mRNA expression of MRP7 in HEK/pcDNA and HEK/MRP7 cells. (A) The effect of 0.3 µM tariquidar treatment on the protein expression of MRP7 in HEK/pcDNA and HEK/MRP7 cells at 0, 4, 12, 24, 48 and 72 h. (B) The effect of 72 h tariquidar treatment at difference concentrations on the protein expression of MRP7 in HEK/pcDNA and HEK/MRP7 cells. (C) mRNA levels of *MRP7* in HEK/pcDNA and HEK/MRP7 cells treated with 0.3 µM tariquidar at different time points. GAPDH was used as a loading control. The protein or mRNA levels of MDR7 were normalized to those of GAPDH and shown on the right. Results are represented as mean ± SD. *P* values were obtained using analysis of variance by comparing the relative amounts of protein or mRNA in cells treated with tariquidar with those in untreated control cells. Results representative of 3 independent experiments are shown. *: *P*<0.05; **: *P*<0.01.

The reversal of MRP7-mediated MDR can be achieved either by decreasing MRP7 expression or by inhibiting its function. To study the effect of tariquidar on MRP7 expression, we treated HEK/MRP7 cells with tariquidar at 0.3 µM for different periods of time (0, 4, 12, 24, 48, and 72 h). Immunoblotting indicated that tariquidar treatment for more than 24 h significantly downregulated the MRP7 protein expression in a time-dependent manner (**Figure 3A**). Treatment of HEK/MRP7 cells with tariquidar at 0, 0.03, 0.1 or 0.3 µM for 72 h also led to downregulation of MRP7 expression in a concentration-dependent manner (**Figure 3B**). RT-PCR was used to ascertain whether the mRNA level of *MRP7* was downregulated in HEK/MRP7 cells after incubation with tariquidar at 0.3 µM. As shown in **Figure 3C**, mRNA levels of *MRP7* did not change significantly in the presence of tariquidar, even after 72 h. These results indicated that tariquidar downregulated MRP7 expression at the post-transcriptional level.

### Tariquidar does not alter the subcellular localization of MRP7

**Figure 4:**
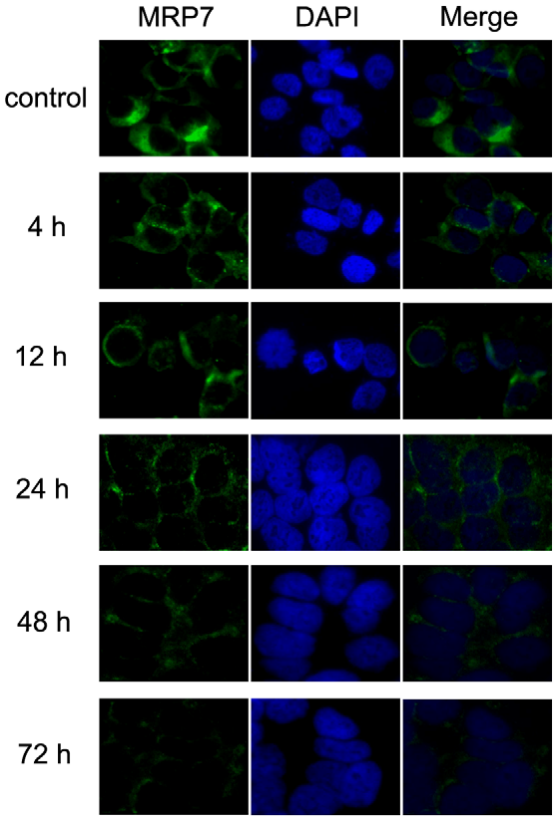
Effect of tariquidar treatment on the subcellular localization of MRP7. HEK/MRP7 cells were treated with 0.3 µM tariquidar for different periods of time. The subcellular localization of MRP7 was analyzed by immunofluorescence. MRP7 staining is shown in green. DAPI (blue) counterstains the nuclei.

Downregulation of MRP7 protein expression can also be accomplished by translocation of MRP7 from the plasma membrane to the cytoplasm or nucleus. To examine whether tariquidar affects the protein location of MRP7, we treated HEK/MRP7 with tariquidar at 0.3 µM for different time points (0, 4, 12, 24, 48, and 72 h) and detected the expression and localization of MRP7. The result of immunofluorescence is shown in **Figure 4**, and there was no alteration of MRP7 protein localization although the expression of MRP7 was downregulated by tariquidar treatment especially up to 24 h treatment of tariquidar at 0.3 µM. This result suggests that tariquidar is able to downregultate MRP7 protein expression but does not alter the localization of MRP7.

### Tariquidar promotes MRP7-mediated intracellular accumulation of paclitaxel

**Figure 5:**
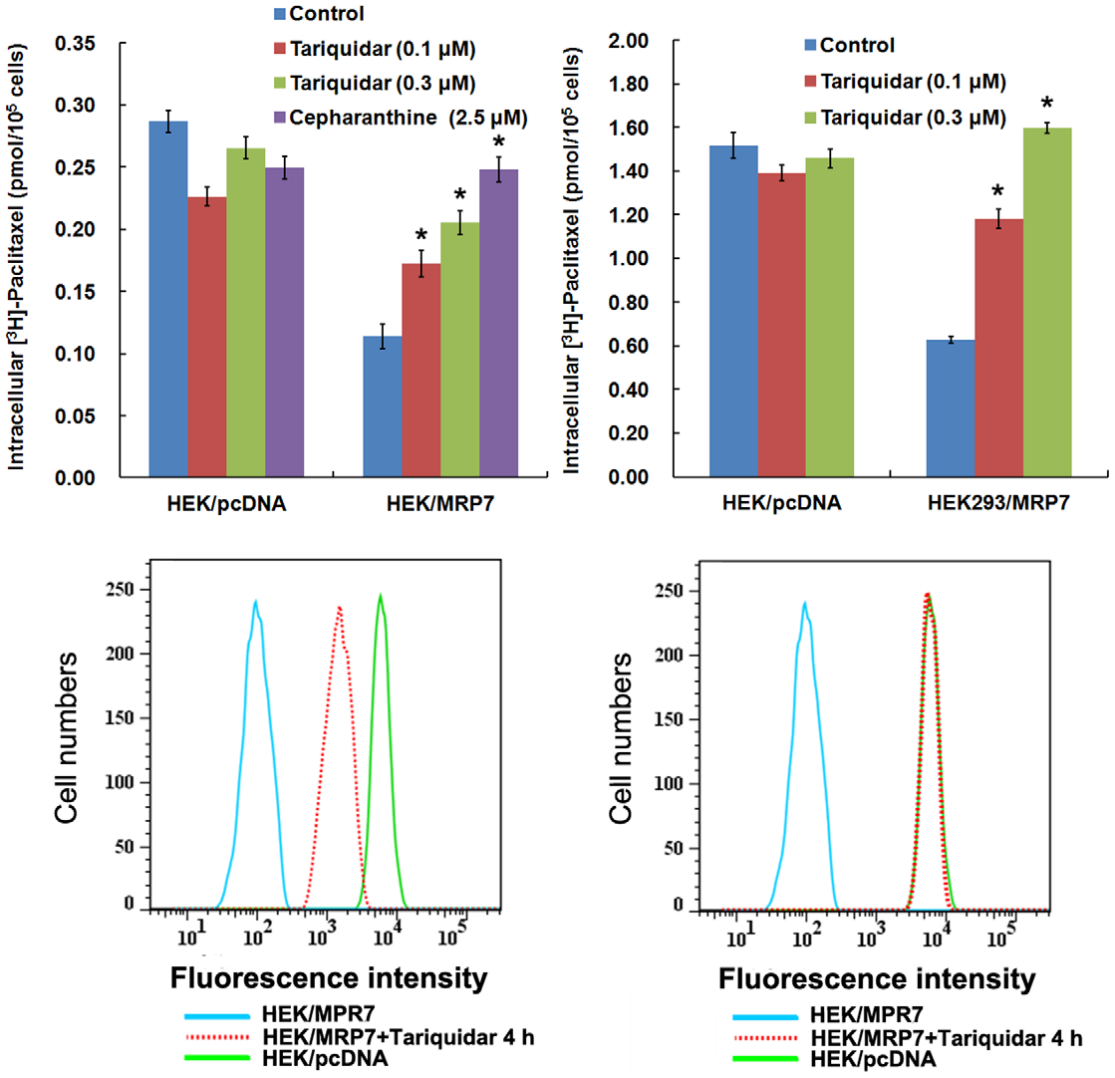
Effect of tariquidar on the accumulation of paclitaxel in HEK/pcDNA and HEK/MRP7 cells. The accumulation of [^3^H]-paclitaxel was measured after preincubation in the presence or absence of tariquidar at 0.1 µM and 0.3 µM for 2 h (A) or 70 h (B) at 37°C, followed by incubation with 0.1 µM [^3^H]-paclitaxel with or without of the reversal agents for another 2 h at 37°C. Then, the cells were collected and the intracellular level of [^3^H]-paclitaxel was detected by scintillation counting. Experiments were done in triplicates, and results were expressed as mean ± SD. *P<0.05, versus the respectively untreated controls. Flow cytemetric detection of the amount of intracellular BODIPY-paclitaxel inside HEK/MRP7 cells after treatment with tariquidar for 4 h and 72 h. The histograms represent the fluorescence signal of intracellular BODIPY-paclitaxel in the presence (dashed line) or absence (shaded histogram) of 4 h (C) or 72 h (D) treatment with 0.3 µM tariquidar.

To determine the effect of tariquidar on the function of MRP7, we measured the accumulation of [^3^H]-paclitaxel in the presence or absence of tariquidar in HEK/pcDNA and HEK/MRP7 cells. The intracellular concentration of [^3^H]-paclitaxel in HEK/MRP7 cells was 39% of that in HEK/pcDNA cells. After 4 h treatment, tariquidar, at 0.1 and 0.3 µM, enhanced the intracellular [^3^H]-paclitaxel accumulation in HEK/MRP7 cells by 1.5 folds and 1.8 folds, respectively (*P*<0.05) (**Figure 5A**). In addition, treatment with tariquidar at 0.1 and 0.3 µM for 72 h increased the intracellular accumulation of [^3^H]-paclitaxel in HEK/MRP7 cells to 1.9 folds and 2.5 folds, as compared to untreated cells (**Figure 5B**). Consistent with our previous observation that tariquidar could downregulate the expression of MRP7 after 24 h, the intracellular concentration of [^3^H]-paclitaxel was higher after 72 h than after 4 h in HEK/MRP7 cells treated with the same concentration of tariquidar (**Figure 5A and 5B**). Of note, tariquidar did not alter the intracellular accumulation of [^3^H]-paclitaxel in the parental HEK/pcDNA cells, suggesting that the action of tariquidar was specific to MRP7.

We further examined the effect of tariquidar on the intracellular accumulation of paclitaxel using BODIPY-paclitaxel, a known fluorescent substrate of MRP7, by flow cytometry. Treatment of 0.3 µM tariquidar for 4 h significantly increased the accumulation of BODIPY-paclitaxel in HEK/MRP7 cells compare to the parental HEK/pcDNA cells (**Figure 5C**). Similar to the above accumulation results, the intracellular retention of BODIPY-paclitaxel was much higher after 72 h tariquidar treatment (**Figure 5D**).

### Tariquidar inhibits the efflux of paclitaxel in MRP7-expressing cells

**Figure 6:**
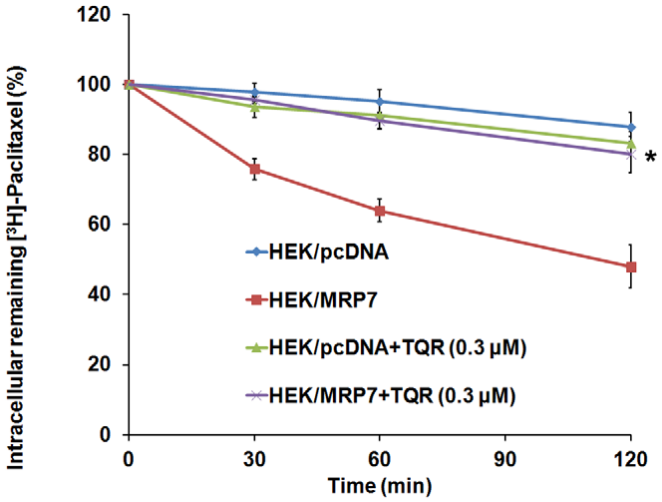
Effect of tariquidar on the efflux of paclitaxel in HEK/pcDNA and HEK/MRP7 cells. The efflux assay was performed as described in Materials and Methods. The values at 0 min were set as 100%. Each data point represents the mean ± SD of three independent experiments, each performed in triplicates. *P<0.05, versus HEK/MRP7 cells without tariquidar treatment.

To investigate whether increased intracellular accumulation of paclitaxel accumulation upon tariquidar treatment resulted from direct inhibition of the efflux activity of MRP7, we performed a time course study to determine efflux of [^3^H]-paclitaxel in the presence of tariquidar. HEK/MRP7 cells, but not the parental HEK/pcDNA cells, rapidly effluxed [^3^H]-paclitaxel, leading to a 50% reduction of intracellular [^3^H]-paclitaxel after 120 min. In the presence of 0.3 µM tariquidar, the efflux of intracellular [^3^H]-paclitaxel was significantly inhibited only in HEK/MRP7 cells but not the parental HEK/pcDNA cells (**Figure 6**). These results indicate that tariquidar inhibited the efflux of paclitaxel by MRP7, leading to increased intracellular accumulation of paclitaxel.

## Discussion

Tariquidar is one of a series of XR compounds initially developed by Xenova Group [25], [26]. It has been reported to achieve a complete reversal of resistance at very low concentrations (25–80 nM) and to have long duration of activity in many murine and human cell lines [27]. The potency of tariquidar is 10- to 30-fold greater than that of the second generation P-gp inhibitors, such as PSC833 [28], and several logs more potent than the first generation of P-gp inhibitors CsA and verapamil [29], [30]. In addition to P-gp, this potent reversal agent was also shown to interact with other ABC transporters in recent studies [31], [32], [33].

Despite the reports showing the widespread tissue expression of MRP7 [34], [35], it remains one of the least characterized ABC family members. MRP7 expression level is upregulated in NSCLC as compared to normal lung tissues, and higher expression is correlated to advanced pathological grades and TNM stages in adenocarcinoma [36]. In hepatocellular carcinoma, MRP7 expression level is also elevated when compared with normal adjacent healthy liver samples [37], and *MRP7* gene expression levels in colorectal tumors correlate with tumor grade (*P* = 0.01) [38]. Absence of this transporter *in vivo* sensitizes animals to taxanes, with *Mrp7* ^−/−^ mice exhibiting increased sensitivity compared to their wild-type counterparts following paclitaxel treatment [12], implying that increased MRP7 expression might be a biomarker for and regulator of treatment response in certain cancers. It has previously been shown that overexpression of MRP7 *in vitro* confers resistance to an unusually wide range of clinically valuable anticancer drugs, including taxanes, vinca alkaloids, nucleoside analogs and epothilone B [9], [11], [39]. Taken together, these findings suggest that modulation of MRP7 activity by inhibitors may have clinical value in the management of certain human cancers.

To our knowledge, this is the first report on the effect of tariquidar on MRP7-mediated MDR. Our data showed that tariquidar could potently reverse MRP7-mediated MDR. Tariquidar significantly sensitized MRP7-expressing cells to a variety of MRP7 substrates, including paclitaxel, docetaxel, vincristine, vinblastine, and vinorelbine. Tariquidar was capable of completely reversing MRP7-mediated MDR at 0.3 µM, making it one of the most potent MRP7 reversal agents identified so far. Consistent with cytotoxicity data, drug accumulation studies showed that tariquidar significantly enhanced the intracellular accumulation of [^3^H]-paclitaxel in MRP7-expressing cells. This result was further confirmed by flow cytometry using the fluorescent MRP7 substrate BODIPY-paclitaxel. Efflux data indicated that the increased intracellular accumulation of [^3^H]-paclitaxel was partly contributed by rapid and direct inhibition of MRP7-mediated drug effux by tariquidar within a short time period (2 to 4 h), as tariquidar-induced downregulation of MRP7 protein expression only occurred after 24 h treatment. Prolonged tariquidar treatment (>24 h) downregulated MRP7 protein expression in a time- and concentration-dependent manner. Therefore, the reversal of MRP7-mediated MDR in HEK/MRP7 cells by tariquidar involved both direct inhibition of MRP7 efflux function and downregulation of MRP7 protein expression. Interestingly, the downregulation of MRP7 protein expression was not accompanied by decreased *MRP7* mRNA levels or altered MRP7 subcellular localization. We therefore speculate that the downregultation of MRP7 protein expression could result from increased protein degradation in response to prolonged tariquidar treatment.

It would be of great interest to characterize the mechanism of regulation of MRP7 expression. It was reported that doxorubicin-stimuli leaded to the expression of MRP7 mediated by a p53-dependent process. Under apoptotic conditions, the *MRP7* mRNA levels remarkably increased in doxorubicin-treated MCF7 cells, whereas its up-regulation was suppressed in p53-dominant-negative MCF7 cells. The level of *MRP7* mRNA was enhanced 54-fold by doxorubicin-induced apoptosis in wild-type MCF7 cells, but only 4.7-fold in p53 dominant-negative MCF7 cells which functionally inactives wild-type p53 [34]. PI3K/AKT is another important pathway which involve in the regulation of ABC transporter expression. EGFR exerts a post-transcriptional enhancing effect on BCRP expression via the PI3K/AKT signaling pathway, which can be attenuated by EGFR inhibitors [40]. Gefitinib and PD158780 reduced both total and surface expression of BCRP in EGFR-positive MDCK BCRP cells by interaction with the PI3K/AKT signaling pathway [40]. PI3K/AKT signaling pathway was also possibly implicated in subcellular localization of BCRP. It was shown that paharmacological inhibition of AKT signaling results in gradual relocalization of BCRP from the EVs membrane to the cytoplasm [41]. In addition, it also has shown that IL-18 and IL-12 together could decrease ABCA1 expression and cellular cholesterol efflux in THP-1 macrophage-derived foam cells through the IL-18R/NF-κB signaling pathway [42]. The molecular mechanism underlying the downregulation of MRP7 expression by tariquidar should be investigated in further studies.

The anticancer activity of tariquidar is currently under wide investigation in preclinical research and clinical trials. In both muriune xenografts and patients, co-administration of tariquidar restored the antitumor activity of paclitaxel [43], docetaxel [44], etoposide and vinorelbine [29], [45], [46]. It was also reported that tariquidar increased the cytotoxicity of doxorubicin and vinblastine in the multicellular tumor spheroid model derived from the MCF7^WT^ breast cancer cell line [47]. The plasma pharmacokinetics of tariquidar has been studied in healthy male volunteers. The peak serum concentration (C_max_) and exposure (AUC) was 2.3 µM and 12.6 µM/h at 2 mg/kg, and the terminal elimination half-life was 26 h [48]. In this study, tariquidar could completely reverse MRP7-mediated MDR at a concentration as low as 0.3 µM, which is an achievable clinical dose. In addition, tariquidar was well tolerated with no dose-limiting toxicities up to 2 mg/kg, and maximum inhibition of P-gp was observed by rhodamine efflux assay and the inhibition persisted for 24 h [48]. In a Phase I clinical trial, tariquidar in combination with vinorelbine in adults with refractory solid tumors was conducted. There were no novel toxicities and no alteration in vinorelbine pharmacokinetics was observed during coadministration of tariquidar [46]. A Phase II study of tariquidar in patients with chemotherapy-resistant advanced breast cancer was conducted. However, the study was terminated, and it was concluded that tariquidar had limited clinical activity to restore sensitivity to anthracyclines or taxanes chemotherapy [49]. Two Phase III trials of tariquidar in combination with chemotherapy were initiated in NSCLC patients. Both of them were stopped due to chemotherapy-related toxicity in the tariquidar arm [50]. It is probable that the toxicity was not due to pharmacokinetic interactions but pharmacodynamic interactions. In one Phase II trial, vinorelbine combined with tariquidar was tolerated at 22.5 mg/m^2^, whereas the starting dose for the Phase III trial was 25 mg/m^2^ combined with tariquidar. The paclitaxel dose in the combination was 200 mg/m^2^, which is somewhat higher than that approved by the FDA for solid tumors. In addition, careful selection of tumor type and cytotoxic agents may also be needed. Reversal clinical trials should include tumor patients that are chemosensitive to the initial therapy but develop drug resistance or tumors that express high levels of ABC transporters based on the tissue of origin. In this study, we found that tariquidar also had effect on MRP7-mediated MDR, thus warranting MRP7 as another factor to be considered in addition to P-gp and BCRP in the chemotherapy resistant patients.

Although most of the clinical trials that investingated the combination of tariquidar with anticancer drugs to improve the therapeutic outcome in cancer patients are closed, tariquidar is still considered as an appropriate compounds for testing the role of P-gp in resistant cancer cells [50]. To date, little information is known about the biology of MRP7, with few MRP7 inhibitors identified and no MRP7 inhibitors evaluated in clinics. Paclitaxel was the substrate of both P-gp and MRP7. However, one recent study showed that *Mrp7* ^−/−^ mice exhibit increased tissue sensitivity and lethality resulting from paclitaxel treatment compared to wild-type counterparts, which indicated that MRP7 functions as a major determinant of taxane sensitivity in mice [12]. So it will be of considerable interest to assess MRP7 protein expression levels in tumors versus normal tissues in the context of treatment with taxanes. More recently, we conducted *in vivo* MRP7 overexpressing tumor xenograft nude mouse model study and found that MRP7 transfected tumors are more resistant to paclitaxel, and this resistance can be reversed by nilotinib, a potent reversal agent for MRP7-mediated MDR [51] Our finding therefore warrants clinical studies to test the efficacy of tariquidar in reversing MRP7-mediated MDR in chemotherapy-resistant patients.
